# A review of brain circuitries involved in stuttering

**DOI:** 10.3389/fnhum.2014.00884

**Published:** 2014-11-17

**Authors:** Anna Craig-McQuaide, Harith Akram, Ludvic Zrinzo, Elina Tripoliti

**Affiliations:** ^1^Imperial College School of MedicineLondon, UK; ^2^Unit of Functional Neurosurgery, Sobell Department of Motor Neuroscience and Movement Disorders, UCL Institute of Neurology, University College LondonLondon, UK; ^3^Victor Horsley Department of Neurosurgery, National Hospital for Neurology and NeurosurgeryLondon, UK

**Keywords:** stuttering, palilalia, speech neural control, basal ganglia, thalamus, deep brain stimulation

## Abstract

Stuttering has been the subject of much research, nevertheless its etiology remains incompletely understood. This article presents a critical review of the literature on stuttering, with particular reference to the role of the basal ganglia (BG). Neuroimaging and lesion studies of developmental and acquired stuttering, as well as pharmacological and genetic studies are discussed. Evidence of structural and functional changes in the BG in those who stutter indicates that this motor speech disorder is due, at least in part, to abnormal BG cues for the initiation and termination of articulatory movements. Studies discussed provide evidence of a dysfunctional hyperdopaminergic state of the thalamocortical pathways underlying speech motor control in stuttering. Evidence that stuttering can improve, worsen or recur following deep brain stimulation for other indications is presented in order to emphasize the role of BG in stuttering. Further research is needed to fully elucidate the pathophysiology of this speech disorder, which is associated with significant social isolation.

## INTRODUCTION

Stuttering (stammering in British English) is a speech disorder characterized by disruptions in speech motor behavior (repeated or prolonged articulatory and phonatory actions) that result in sound and syllable repetitions, audible and inaudible sound prolongations and broken words (Max et al., 2004b). The definition of stuttering remains the subject of debate, despite multiple attempts (Andrews and Harris, 1964; Wingate, 1997; Bloodstein and Ratner, 2008). For the purposes of this review stuttering will be considered a speech motor disorder, even if the process may have broken down at the pre-motor level and even if there is a cognitive/linguistic or emotional/psychological processes related. Stuttering has a negative impact upon quality of life, interpersonal relationships, employment opportunities and job performance, and it is associated with significant personal financial costs (Klein and Hood, 2004; Blumgart et al., 2010; Koedoot et al., 2011; Van Borsel et al., 2011). Stuttering is associated with stigma and discrimination due to negative stereotypes, especially if severe and if causality is perceived to be psychological (Gabel, 2006; Boyle et al., 2009). It is associated with higher levels of social anxiety (Kraaimaat et al., 2002; Iverach et al., 2009a,b). Although ∼1% of the population stutters (Van Riper, 1982), the etiology is still unknown and a unifying pathomechanism for acquired neurogenic stuttering (ANS) has yet to be identified. The aim of this review is to describe neuroimaging, lesion, pharmacological, and genetic studies on the neural circuitries implicated in developmental and acquired stuttering.

## PERSISTENT DEVELOPMENTAL STUTTERING

Persistent developmental stuttering (PDS) often first manifests in children between the ages of 2 and 4. It improves or remits spontaneously in a large proportion of affected children, boys having a much higher rate of persistence into adulthood than girls. Stuttering can also occur *de novo* in adulthood secondary to neurological injury or disease. However resolved childhood stuttering can recur in the context of adult onset neurological disease such as Parkinson’s disease (PD). Despite an extensive literature on the subject, the etiology of PDS remains unknown.

### FEATURES OF DEVELOPMENTAL STUTTERING

Repetition of sounds, syllables, and words, prolongation of sounds and blocks in speech are three classical features of stuttering. PDS typically occurs predominantly at syllable initial position or word initial position. PDS is said to show adaptation (decreased dysfluency with repeated reading of same passage) and consistency (tendency for stuttering to recur in the same words/syllables in successive readings of the same text; Bloodstein and Ratner, 2008). However, some are very critical of the “adaptation effect” and “consistency effect” (Wingate, 1997).

### FLUENCY-INDUCING CONDITIONS

Dysfluency in patients with PDS is typically said to improve with certain ‘fluency-inducing conditions’ (Bloodstein and Ratner, 2008). The improvement in stuttering with fluency-inducing conditions suggests that the pathology affects the CNS at a speech motor planning level rather than being due to abnormalities of the vocal tract or of the peripheral nervous system. Fluency-inducing conditions include choral speech or reading, the rhythm effect (or metronome speech), non-automated speech (e.g., foreign accent, role play, or acting), white noise, and singing (Stager and Ludlow, 1998; Kalinowski et al., 2000; Kalinowski and Saltuklaroglu, 2003; Davidow et al., 2011).

Altered auditory feedback, including delayed auditory feedback (DAF) and frequency altered auditory feedback (FAF), can temporarily induce fluency in persons who stutter (PWS; Kalinowski et al., 1993; Macleod et al., 1995; Stuart et al., 1996, 2008).

### ASSOCIATED SYMPTOMS

Associated features or “secondary” symptoms can be divided into overt concomitants and physiological concomitants (Bloodstein and Ratner, 2008). Overt concomitants include associated movements which may be due to underlying motor dysfunction (e.g., visible tension in the face, head jerking while speaking, eye blink, forehead wrinkling, sudden exhalation), and interjected speech fragments or ‘filled pauses,’ which can be sounds, syllables, words, or phrases. There may also be abnormal speech rate and altered vocal quality, with sharp shifts in pitch level or lack of normal pitch variation. Physiological concomitants include flushing, pallor, perspiration, eye movements, and cardiovascular phenomena.

## PUTATIVE GENETIC ETIOLOGIES OF PDS

The aggregation of PDS in certain families, high rates of monozygotic (63%) and dizygotic twin concordance, as well as reports of significant difference in sex ratio between stutterers with and without a positive family history have led to extensive research into a potential genetic etiology of the disorder (Howie, 1981; Drayna et al., 1999). No single gene has been identified in PDS, and it is likely a polygenic disorder. There is evidence to suggest a Mendelian model with an autosomal dominant major gene effect (Viswanath et al., 2004). An area on chromosome 18 was identified in a genome-wide linkage analysis of stuttering (Shugart et al., 2004). This area was relatively large, but putative candidate genes included a cluster of genes belonging to the desmoglein/desmocolin family (on 18q12.1), and the neuronal cadherin gene 2 (on 18q11.2). Both have known roles in cell adhesion and intercellular communication, and might be of relevance to neural substrates of speech. The results of other genome-wide linkage surveys suggest linkage on chromosomes 1, 13, and 16 (Cox and Yairi, 2000), and on chromosome 12q (study of 46 consanguineous families, Riaz et al., 2005). Mapping of the significant locus on chromosome 12q identified mutations in three related genes implicated in lysosomal metabolism. A link between these mutations in lysosomal metabolism genes and the white matter (WM) abnormalities described in PWS has been suggested (Büchel and Watkins, 2010).

Watkins (2011) makes a comparison of stuttering with a genetic disorder of speech and language development described in the large multigenerational KE family, which displays autosomal dominant monogenic inheritance. The affected members of the KE family have been found to have a mutation in the FOXP2 gene in the SPCH1 region of chromosome 7q31 (Lai et al., 2001). The chromosome 7 locus identified by Suresh et al. (2006) did not include the FOXP2 gene. Voxel based morphometry (VBM) and positron emission tomography (PET) studies of affected KE family members found structural and functional abnormalities of the caudate nucleus (Watkins et al., 1999, 2002; Watkins, 2011), and FOXP2/Foxp2 is expressed in the dorsal striatum in human and rat embryogenesis. FOXP2 is also expressed in a homologous area of the songbird brain and knockout of the gene in songbirds is associated with severe impairment of song learning, with stuttering-like output (Haesler et al., 2004, 2007). Thus the genetic and neuroimaging findings in the KE family provide evidence of a possible genetic ontogeny to stuttering. The structural and functional abnormality of the caudate supports the hypothesis that stuttering is a basal ganglia (BG) disorder, and is consistent with certain neuroimaging studies in stuttering (see below).

Alm and Risberg (2007) suggested that the adult stutterers in their study could be divided into two groups, the first comprising those with higher trait anxiety and higher Wender Utah Rating Scale (WURS) scores. The WURS is used in the retrospective diagnosis of childhood attention deficit hyperactivity disorder (ADHD). The stutterers in this group had a higher occurrence of pre-existing neurological lesions or had relatives who stuttered. In contrast, the stutterers in the second group had lower trait anxiety and WURS scores, fewer pre-onset neurological lesions, and more relatives who stuttered. They thus posited that these groups might represent two separate subtypes of stuttering.

This is consistent with Poulos and Webster (1991), who suggested that adults with developmental stuttering can be divided into two groups, one with a family history of stuttering and therefore possible genetic etiology, and another with no family history of stuttering but a history of pre-onset head injury or birth injury. There is evidence of a relationship between mild head injury and stuttering (and hyperactivity and mixed handedness) in children (Segalowitz and Brown, 1991).

## NEURAL CORRELATES OF PERSISTENT DEVELOPMENTAL STUTTERING

**FIGURE 1:**
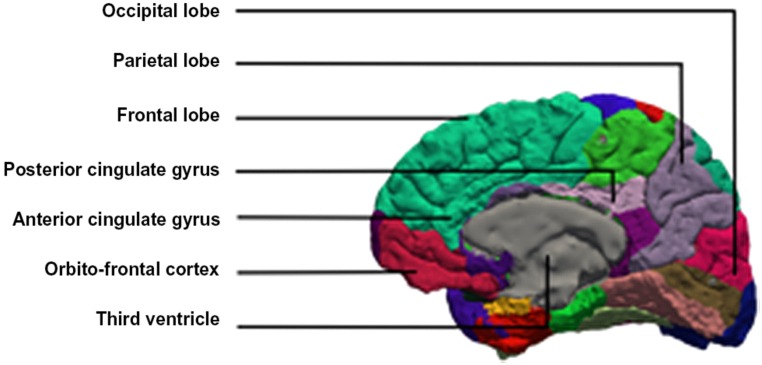
**Medial view of the brain with the anterior and posterior cingulate gyrus**.

Brain imaging studies of developmental stuttering have disclosed various abnormalities. In this paper we discuss evidence for the role of the cerebellum, the anterior cingulate cortex (ACC), the supplementary motor area (SMA), and the right frontal operculum (RFO) (**Figure 1**).

### THE CEREBELLUM AND AUDITORY PROCESSING

The cerebellum has classically been considered to be a motor structure implicated in motor learning and in novel behaviors. A meta-analysis of functional imaging literature showing that it is consistently activated in purely auditory tasks suggests that it might also have a role in sensory auditory processing (Petacchi et al., 2005).

There is evidence of greater overall cerebellar activation and abnormal right lateralization in stutterers compared to controls during silent and oral word reading, which increases further following fluency-inducing therapy but then falls to below pre-treatment levels in the long term (De Nil et al., 2001). Increased cerebellar activation in PWS compared to controls both pre- and post-treatment may be related to increased sensory or motor monitoring due to reduced automaticity in articulatory movement sequences, even when reading silently (De Nil et al., 2001).

Cerebellar activation may also be related to selected attention processes, and prior treatment in stutterers may lead to greater attention and monitoring during speech production and thus less automation in articulatory movement execution (Allen et al., 1997). The increase in cerebellar activation from pre- to post-treatment followed by a decrease in activation would be consistent with this hypothesis as speech therapy would initially reduce automaticity and increase self-monitoring and attentional effort during speech and this would then decrease as the acquired skills for fluency became more practiced and automatic with time.

**FIGURE 2:**
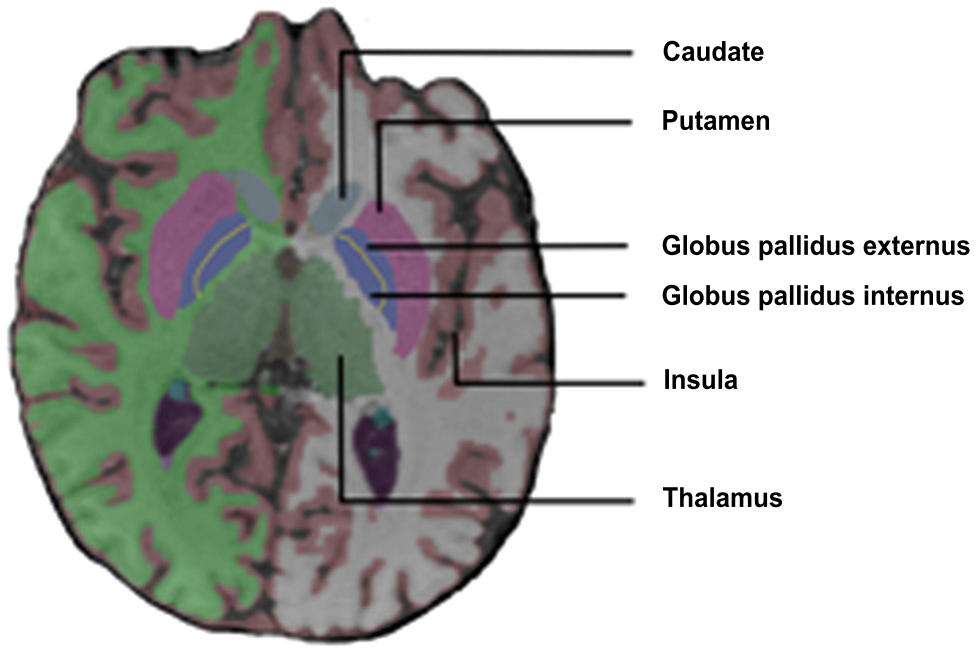
**Coronal view of the basal ganglia**.

Fox et al. (1996) reported a diffuse increase in activation of the cerebral and cerebellar motor systems in stutterers [M1, SMA, superior lateral premotor region (SLPrM), and cerebellum] during solo and chorus reading conditions. The M1 activation was aberrantly right dominant in the dextral stutterers. They also found that stutterers (but not controls) activated the insula bilaterally and the claustrum, the thalamus and the globus pallidus (GP) on the left during speech tasks (**Figure 2**).

### THE ANTERIOR CINGULATE CORTEX AND THE SUPPLEMENTARY MOTOR AREA IN STUTTERING

A review of the neural pathways underlying vocal control found evidence for divergent roles of the anterior cingulate gyrus in human and non-human primates (Jürgens, 2002). Studies in macaques report vocalization-correlated activity changes in anterior cingulate gyrus neurones, whereas human PET and functional magnetic resonance imaging (fMRI) studies show that the SMA is consistently activated during speech and singing. The anterior cingulate gyrus is only activated during a few speech-related tasks in humans, but shows consistent activation during non-vocal emotional-related tasks. Thus it may be that the SMA is implicated in the volitional control of learned motor patterns, and the anterior cingulate gyrus in the volitional control of emotional states. A unifying feature is the control of initiation of vocal utterances rather than pattern generation.

Functional imaging studies of stuttering in humans provide evidence for the implication of the ACC in atypical neural activation patterns during speech, with relatively increased activation in stutterers in the ACC during silent and oral reading tasks (Kroll et al., 1997a,b; De Nil, 1999b). De Nil and Kroll (2001) proposed that the ACC provides a connection between the limbic system and the sensorimotor cortex of direct relevance to stuttering. The ACC is involved in response preparation and in anticipatory reactions, particularly when presented with complex stimuli and the need to select one of multiple possible responses (Paus et al., 1998). Thus increased ACC activation in stutterers might be due to increased anticipatory reactions when reading and scanning for potential fluency problems, and the ACC could also be involved in the silent rehearsal of words (De Nil and Kroll, 2001). Less automated tasks are associated with increased activation of the inner articulatory loop, which may involve the ACC (Paus et al., 1993). Furthermore, ACC activation during silent reading tasks is significantly decreased in stutterers following fluency-inducing treatment (Kroll et al., 1997a,b; De Nil, 1999a,b). This could be due to decreased silent articulatory rehearsal or decreased anticipatory scanning.

PET and fMRI studies have shown consistent activation in the sensorimotor cortex, the SMA and the anterior cingulate gyrus during speech and singing in humans. There are other areas that show task-specific activation. Pronouncing sequences of meaningless phonemes is associated with activation in the insula and auditory cortex (Bookheimer et al., 2000), whereas passive listening to external sounds and auditory feedback of produced sounds activate the auditory cortex but not the insula (Perry et al., 1999). Furthermore, activation of the insula is seen during singing and speaking aloud but not during silent speech and song nor when listening to speech or tone sequences (unlike the auditory cortex; Herholz et al., 1994; Riecker et al., 2000).

### AUDITORY FEEDBACK AND STUTTERING

There is evidence of voice-sensitive and –selective clusters of activation in the superior temporal sulcus (STS), which may be analogous to face-selective areas in human visual cortex (Belin et al., 2000). The association of increased superior temporal cortex activation with mismatch between actual and expected auditory feedback lends support to a verbal self-monitoring model, in which there is communication between speech production regions and speech perception regions (Fu et al., 2006). This is consistent with activation in the superior temporal gyrus (STG) bilaterally for DAF conditions compared to normal auditory feedback. The positive correlation between DAF and STG activation suggests that areas of the temporo-parietal cortex are the substrate for a conscious verbal self-monitoring that supports automatic speech production (Hashimoto and Sakai, 2003).

A magnetoencephalography (MEG) study of cortical activation in response to hearing one’s own voice showed that the human auditory cortex is primed by speech at a millisecond rate and that this delays and decreases reactions to one’s own expected vocal output (Curio et al., 2000). Stuttering could involve abnormalities in this type of motor to sensory priming of auditory cortex responses during speech output (Salmelin et al., 1998). The sensitivity of the auditory cortex is reduced when listening to one’s own voice, and the activity of the auditory cortex may be modulated according to predicted or expected auditory feedback (Houde et al., 2002).

Riecker et al. (2002) found that a rhythmic speech production task was associated with activation of the left putamen and thalamus and right perisylvian areas, including STG and right Broca analog, and propose that the right hemisphere is implicated in rhythmic pattern rehearsal and the left hemisphere in self-monitoring of verbal output.

A MEG study of PWS’ auditory cortex response in tasks with and without auditory feedback suggested an unstable interhemispheric balance in stutterers, easily disturbed by increased workloads, which could lead to unpredictable and transient failure of auditory perception (Salmelin et al., 1998). Accurate interpretation of auditory input is needed for self-monitoring and on-line speech adjustment, therefore this abnormal auditory response could initiate or facilitate stuttering.

Previous studies suggested that a significant proportion of stutterers fail to show the normal right ear advantage in dichotic presentation of meaningful linguistic stimuli (Curry and Gregory, 1969; Hall and Jerger, 1978), and that stutterers may even have difficulties in sound localization (Rousey et al., 1959).

Braun et al. (1997) used PET to investigate speech in adults who stuttered since childhood. They found that rCBF patterns in stuttering differed markedly from normal controls, failing to demonstrate left hemispheric lateralization typically observed in controls; instead regional responses were either absent, bilateral or localized to the right. During a dysfluent language task, stutterers had increased right caudate, bilateral periaqueductal gray (PAG) and midline cerebellar activations. The dysfluent language-motor contrast also showed absence of left inferior insular cortex activation in stutterers compared to controls. There was disproportionate activation of anterior forebrain regions (which have a regulatory role in motor function) in stutterers during dysfluent speech production, and at the same time, a relative deactivation of post-rolandic regions involved in sensory information perception and decoding. In the dysfluent language-motor contrast, stutterers failed to activate Wernicke’s area (posterior STG and inferior angular gyrus). Thus when dysfluent, stutterers may not be effectively monitoring speech language output, as this is the role of Wernicke’s area in normally fluent individuals (Petersen et al., 1988; Wise et al., 1991; Démonet et al., 1992; Zatorre et al., 1992). Van Borsel et al. (2003a) also reported increased activation of the right homologs of left language areas in stutterers during language processing, as well as increased cerebellar and auditory activations during silent reading, which may demonstrate the use of less differentiated auditory and motor feedback mechanisms in stutterers, and might partly explain the fluency-enhancing effects of various types of AAF in PDS.

Conversely, Ingham et al. (2003) found only two regions with different activation between PWS and controls, namely increased activation of the right anterior insula and deactivation of right Wernicke’s homolog (BA21/22) in stutterers. This differs from the results of Pool et al. (1991), who report global absolute reductions in rCBF in PWS compared to controls, with significant flow asymmetry (left < right) in the anterior cingulate and the superior and middle temporal gyri in PWS. Ingham et al. (1996) failed to find any significant differences in resting state regional cerebral perfusion between PWS and controls (on PET and MRI), but rather only suggested minor differences in hemispheric symmetry, despite adequate sample size (*n* = 29), 74 regions of interest and sound methods. The discordance of results between these studies may illustrate the lack of consensus in the neuroimaging literature and motor speech research.

### NEURAL ACTIVATION CHANGES FOLLOWING FLUENCY-INDUCING THERAPY

De Nil et al. (2003) provided evidence using PET that fluency-inducing treatment may be associated with a general reduction in overactivation, especially in the motor cortex, and with changes in activation lateralization. There were significant differences in activation patterns between controls and stutterers, even during silent speech tasks (so presumably not attributable to articulatory movements). During silent reading, controls activated speech and language areas in left frontal and temporal cortices, and no activation in motor or premotor cortex. Stutterers showed a significantly increased level of overall neural activation compared to controls, consistent with the hypothesis that in stutterers there is recruitment of more neural resources in order to achieve even relatively simple speech tasks (De Nil et al., 2000). Stutterers activated the primary motor cortex and the cerebellum, even during silent reading tasks, suggesting that they place more emphasis on articulatory aspects even during silent reading (De Nil et al., 2000, 2001). Following treatment, the activation pattern in stutterers became more left-lateralized, only to return to bilateral or right-lateralized at follow-up scanning. There was a gradual reduction in overall activation following treatment and at follow-up. Activation in the insula changed from being predominantly right-lateralized pre-treatment to being left-lateralized post-treatment and at follow-up, whereas cerebellar activation became more right-lateralized with treatment (as expected given its cross-connections to the motor cortex). Pre-treatment, stutterers had right-lateralized STG activation, which became left-lateralized post-treatment and at follow-up.

The neural overactivation seen in stutterers compared to controls during speech-related tasks, and increased cerebellar activation (De Nil et al., 2001) suggests a lack of speech automatization. The results of De Nil et al. (2003) suggest that this aberrant neural overactivation can be reduced in stutterers by fluency-inducing therapy, however, activity was not entirely normalized after treatment. Furthermore, it is not yet known whether neural activation patterns and treatment-induced changes in them differentiate stutterers who will relapse following treatment from those who will successfully maintain fluency in the long term.

Giraud et al. (2001) found that in normal controls the posterior superior temporal cortex (BA42) has a greater PET response to complex sounds than to white noise and Wernicke’s area (BA22) responds specifically to speech sounds, and in cochlear implant patients this specialization of function is absent in both areas. They argue that this demonstrates experience-dependent changes in the functional specialization of the language network thanks to underlying neuroplasticity. Such neuroplasticity in these cortical speech areas could, at least in part, explain changes in neural activation in stutterers following fluency-inducing therapy.

### THE RIGHT FRONTAL OPERCULUM

The RFO has been reported to be the only region consistently overactivated in stutterers compared to controls during both reading and passive semantic decision tasks (Preibisch et al., 2003). The RFO is the right homolog of Broca’s area and could compensate for aberrant transmission between Broca’s area and left motor cortex representations of the larynx and tongue. Consistent RFO overactivation, negatively correlated with stuttering severity, suggests a compensatory overactivation rather than a primary dysfunction (Neumann et al., 2003; Preibisch et al., 2003). Watkins et al. (2008) reported two areas of overactivation in the right anterior insula close to the RFO. There is diffusion tensor imaging (DTI) evidence of decreased fractional anisotropy (FA) in the WM underlying the left rolandic operculum (LRO), an area corresponding to the left sensorimotor representation of the larynx and tongue (BA43; Sommer et al., 2002). Decreased FA suggests demyelination or loss of organization of WM tracts. However, the results reported by Sommer et al. (2002) should be interpreted with caution since the large voxel size used means that WM analysis may be influenced by adjacent gray matter.

Watkins et al. (2008) identified decreased FA of the WM underlying ventral premotor and motor cortical areas which were underactive on fMRI. This area of bilaterally decreased FA was located close to that reported in the LRO by Sommer et al. (2002). The results of Watkins et al. (2008) may suggest decreased WM integrity in tracts, which are important for execution of articulatory movements (via connections with primary motor cortex) and for integration of articulatory planning and sensory feedback (via connections with posterior superior temporal and inferior parietal cortex). The finding of reduced FA in WM underlying the LRO also corroborates the finding of atypical gyral anatomy in stutterers in the same area (Foundas et al., 2001).

In another study, the right middle frontal cortex was the only area of decreased activation in PWS following therapy (Neumann et al., 2005). There was increased activation in an extended network of mainly left-sided areas following therapy, and also in areas of temporal cortex bilaterally. The left insula and the LRO, close to the area of decreased FA in Sommer et al. (2002), both showed increased activation after therapy.

Cases of recovery from aphasia following frontal injury suggest that the right inferior prefrontal cortex can be rapidly activated to compensate for damage to Broca’s area (Heiss et al., 1999; Rosen et al., 2000), and the RFO may be recruited to compensate for left frontal cortex dysfunction in dyslexia (Pugh et al., 2001).

Persons who stutter may have subtle changes in right perisylvian cortical anatomy corresponding to areas of increased activity reported in imaging studies, with an increased number of sulci in the suprasylvian gyral banks and of sulci connecting to the second segment of the right Sylvian fissure (Cykowski et al., 2008). This study failed to find any differences in asymmetry between stutterers and controls in the number of sulci and gyral banks in the left perisylvian language region and the planum temporale.

Reduced WM integrity in the left superior longitudinal fasciculus (SLF) was reported in study of children with developmental stuttering (Chang et al., 2008). The cortex overlying the left SLF includes the rolandic operculum (BA 43), consistent with the area of reduced left hemisphere FA and functional underactivity (Sommer et al., 2002; Watkins et al., 2008).

Using an augmented VBM technique, Jäncke et al. (2004) found that stutterers had increased white matter volume (WMV) in the perisylvian language areas, and atypical anatomy and lateralization in the perisylvian language areas and also in prefrontal and sensorimotor cortex. Stutterers had increased right hemisphere WMV in the STG including the PT and Heschl’s gyrus, in the ITG including the pars opercularis (part of the right-sided homolog of Broca’s area), in the precentral gyrus (M1) including parts of the face, mouth, and hand representations, and in the anterior MFG. The dextral controls had a leftward asymmetry of auditory cortex WM, consistent with the findings of Penhune et al. (1996). Stutterers had symmetric auditory cortex WM volumes. There was no correlation between stuttering severity and the anatomical findings. Jäncke et al. (2004) posited that regional increases in right hemisphere WMV could be due to increased or atypical interhemispheric communications, and that there may be altered processing strategies in the right hemisphere in stutterers.

It should, however, be noted that despite concordant WM FA changes reported in these studies, Connally et al. (2014) described a much more complex picture, with stutterers having significantly decreased WM FA compared to controls in multiple areas. They used diffusion-tensor imaging in a sample of 29 PWS in order to replicate previous findings of the literature that showed reduced integrity in WM underlying ventral premotor cortex, cerebral peduncles and posterior corpus callosum. They also showed that within the group of PWS the higher the stuttering severity index, the lower the WM integrity in the left angular gyrus, but the greater the WM connectivity in the left corticobulbar tract.

A parametric performance correlation analysis of PET rCBF during solo reading and chorus reading found that dextral stutterers had increased activation of the SMA mouth area, but the location and right lateralization of the area were comparable to that in controls (Fox et al., 2000). In the stutterers, the primary motor cortex (M1) was not readily differentiable from the ILPrM (BA44/46, Broca’s area). There was a significant increase in cerebellar activation in stutterers, and this activation was abnormally left-lateralized. Ingham et al. (2000) suggested that the significantly greater cerebellar syllable correlates in stutterers compared to controls and the state effect (stuttering in solo condition) indicate that the cerebellum may play a role in enabling fluent speaking in PDS speakers (in the chorus condition). Stutterers also showed abnormally left-lateralized STG activation.

A gender replication study of the Fox et al. (2000) analysis found that dextral female stutterers had increased activity in the right anterior insula and decreased activity in the left IFG and in right BA21/22, as observed in males (Ingham et al., 2004). In addition to this, female stutterers had activations in the BG (the left GP and the right caudate) and in the left anterior insula. Female stutterers also had widespread deactivation in the right hemisphere (limbic and parietal lobes and prefrontal area). Overall, stutter rate correlated positively with bilateral regional activations in females, and with right-lateralized regional activations in males. There may be a relationship between these gender differences in neural activation and the higher rate of childhood recovery from developmental stuttering in females compared to males.

In an activation likelihood estimation meta-analysis of neuroimaging studies of PDS, word reading in fluent controls was associated with activation in M1, premotor cortex, SMA, rolandic operculum, auditory areas, and lateral cerebellum (Brown et al., 2005). There was considerable overlap between the Talairach co-ordinates of the activations in motor cortex, cerebellum, SMA, and auditory cortex between Brown et al. (2005) and Turkeltaub et al. (2002). These two meta-analyses did not have any studies in common, so it is plausible that these activations represent a set of core areas for speech production. Thus there is a set of areas consistently activated in speech production, namely M1, SMA, premotor cortex, anterior insula, frontal and rolandic opercula, cingulate, quadrangular lobule of the cerebellum and the GP and putamen. These areas may be generally implicated in voluntary vocalization because they are also activated during wordless singing (Perry et al., 1999; Riecker et al., 2000; Brown et al., 2004). In PWS compared to controls, there was a greater number of more widespread areas activated for the same task. Key differences in PWS included overactivation of motor areas (M1, SMA, cerebellar vermis, cingulate), atypical right lateralization of activity in rolandic and frontal opercula and anterior insula, and absence of auditory activations associated with self-monitoring of speech. This is consistent with deactivation in right auditory association cortex and atypical right anterior insula/frontal operculum activation reported by Ingham (2001), Ingham et al. (2004). The function of the RFO/anterior insula has yet to be fully elucidated, but it has been implicated in the processing of vocal fundamental frequency and of prosody (Perry et al., 1999; Riecker et al., 2000; Brown et al., 2004; Meyer et al., 2004; Wildgruber et al., 2004; Hesling et al., 2005). Interestingly, this area is also implicated in Tourette syndrome (Stern et al., 2000).

Stutterers failed to show the, albeit weak, left GP activation of fluent controls, or the activation in any other BG nucleus, so the results of the meta-analysis do not either strongly support or disagree with the BG model of stuttering (Alm, 2004).

Brown et al. (2005) proposed that their findings could be explained by the phenomenon of efference copy, or feedforward projection of a motor plan, in which an inhibitory signal is projected to the perceptual region from the motor region (Numminen and Curio, 1999; Curio et al., 2000; Houde et al., 2002; Leube et al., 2003; Max et al., 2004a). If stuttering is predominantly a problem of motor program initiation, it is plausible that perceptual prediction of speech sounds, an inhibitory signal, is repeatedly delivered to the auditory system, causing word or syllable repetition. Thus efference copy could account for the absence of auditory activation in stutterers (associated with vocal self-monitoring in fluent subjects). In efference copy, there is self-monitoring comparing the expected and actual output, a function in which the cerebellum is believed to have a role (Blakemore et al., 2001). Brown et al. (2005) thus propose that cerebellar overactivation in stuttering may be associated with the discrepancy signal generated from the difference between expected speech output (left auditory cortex) and the actual speech output (right motor cortex), and that their efference copy hypothesis predicts an inverse relationship between the left auditory cortex and the right anterior insula.

Max et al. (2004a) proposed a hypothesis regarding putative sensorimotor etiologies for stuttering. Stuttering may be caused by insufficiently activated or unstable internal models within feedforward and feedback speech movement control subsystems. Thus speech system instabilities in stuttering result from an over-reliance on afferent feedback that has inherent time lags (compared to efference copy or feedforward control). Civier et al. (2010, 2013) has simulated this hypothesis, (i.e., that over-reliance on feedback control leads to production errors which if the grow large enough can cause the motor system to “re-set” and repeat the current syllable), using computer simulations of a “neutrally impaired” version of the DIVA model (Directions Into Velocities of Articulators), a neural network model of speech acquisition and production (Bohland et al., 2010). Simulation results support findings from neuroimaging on the WM disruptions and elevated dopamine levels for PWS.

Watkins et al. (2008) found that both controls and stutterers showed activity in areas including the left IFG, ventral premotor cortex, SMA, pre-supplementary motor cortex and cingulate motor cortex, face sensorimotor cortex, STG and STS and left thalamus and anterior cerebellum during speech production and perception. Overactivation in stutterers compared to controls in the cerebellum, midbrain and anterior insula bilaterally, and underactivation in sensorimotor, ventral premotor, rolandic operculum cortical areas bilaterally and Heschl’s gyrus on the left (Watkins et al., 2008) is consistent with the findings of Brown et al. (2005). Watkins et al. (2008) also report overactivation in stutterers in the midbrain, involving the substantia nigra (SN) as well as the pedunculopontine nucleus, the subthalamic nucleus (STN) and the red nucleus, consistent with BG network dysfunction or abnormalities of dopamine in PWS (see below).

Chang et al. (2008) used VBM and DTI to investigate brain anatomy differences between children who stuttered, children recovered from developmental stutter and normal controls. They showed decreased gray matter volume (GMV) in the right cingulate gyrus in children with persistent stuttering compared to those who had recovered. There was decreased GMV in bilateral MTG/STG, bilateral precentral gyri (BA6) and bilateral cerebellar regions in recovered versus persistent stuttering children. There were also differences in integrity of WM underlying the LRO in children with persistent/recovered stuttering compared to fluent controls. This is consistent with the results of Sommer et al. (2002), and suggests reduced FA in left WM corresponding to motor control of oral articulators.

Thus the results of Chang et al. (2008) suggest a possible association of deficiencies in left hemisphere GMV and decreased left speech system WM integrity with risk of PDS. Chang et al. (2008) did not find any differences in left-right hemisphere asymmetries between stuttering children and controls, nor any increase in right hemisphere speech regions (contrary to other studies on adults with PDS). In the context of PDS in adults, neuroplasticity during development may be implicated in these differences.

In a MEG study of single word reading, Salmelin et al. (2000) found differences in activation sequence, lateralization of neuronal processing, and functional connectivity in relevant motor cortical areas between dextral stutterers and controls. Following visual word presentation, controls showed left inferior frontal cortex activation within 400 ms, which may correspond to articulatory programming or encoding. Subsequently there was activation of the left lateral central sulcus and of the dorsal premotor cortex, corresponding to motor preparation. In stutterers, there was a reversed activation sequence, with early left motor cortex activation and later left inferior frontal activation. Salmelin et al. (2000) thus suggested that stutterers initiate motor programs before articulatory code preparation. Furthermore, stutterers failed to show the left motor and premotor cortex activation seen in fluent controls during word reading tasks. With regards to the suppression of motor cortical 20 Hz rhythm (a MEG correlate of task-related neuronal processing), stutterers showed a right hemisphere dominant response, whereas the controls showed a left dominant response (as expected in dextral subjects).

This is consistent with PET studies showing higher rCBF in right rolandic areas in stutterers compared to controls (Fox et al., 1996; Braun et al., 1997). Thus during speech production in stutterers, the right frontal cortex is very active but fails to produce synchronous time-locked responses. Salmelin et al. (2000) proposed that this failure to produce time-locked responses could be associated with difficulties in initiating the correct prosody in propositional speech in stutterers. The 20 Hz suppression was greatest in the mouth area in controls, but in the hand and mouth areas in stutterers (there were no overt hand movements during the task). This could be a reflection of imprecise functional connectivity between adjacent mouth and hand motor cortex representations in stutterers when speaking.

The findings of Salmelin et al. (2000) support the idea of bilateral cortical abnormalities in stutterers, consistent with the results of neuroimaging results (Wu et al., 1995; Fox et al., 1996; Braun et al., 1997), and suggest dysfunction throughout a bilateral language network, with abnormal timing relationships between premotor and primary motor regions in left hemisphere affecting articulatory and motor preparation for speech and generation of correct prosody. However, the suggestion by Salmelin et al. (2000) that stutterers initiated motor programs inappropriately early before articulatory code preparation was not borne out in other studies which failed to find any clear evidence of problems in assembling speech production motor plans in stutterers compared to controls (Van Lieshout et al., 1996a,b).

There is evidence that PWS have abnormal neural activation patterns in non-speech vocal motor tasks as well as during speech tasks, and the functional abnormalities in PDS may therefore not be limited to speech. During speech and non-speech vocal motor tasks, stutterers consistently showed underactivation of frontal and temporal areas, including the left STG and the left pre-motor cortex (BA6) during perception and planning, and underactivation of the right STG, and of Heschl’s gyrus, the precentral motor region (BA4), the insula and the putamen bilaterally (Chang et al., 2009).

Evidence of increased right hemisphere activation not only during speech production but also during other tasks suggests that increased right hemisphere activation may be inherent in adults who stutter (Preibisch et al., 2003).

Stutterers have atypical neural functions even in the absence of overt speech production during silent reading and event-related brain potentials (ERPs) in stutterers compared to controls suggest differences in functional neural organization and altered processing common to word classes (Weber-Fox, 2001).

## ACQUIRED NEUROGENIC STUTTERING AND SUBCORTICAL BRAIN LESIONS

The published reports indicate that the dominant feature of neurogenic stuttering is repetitions of sounds or syllables, sometimes together with sound prolongations, but blocks with struggle seem to be less common in ANS (Alm, 2004).

In terms of localization of lesions, developmental stuttering (PDS) is associated with a reduction in the WM anisotropy situated just below the left sensorimotor cortex (Sommer et al., 2002), which corroborates the more general observation that the perisylvian region is anatomically more heterogenous in people who stutter than in controls (Foundas et al., 2001). In contrast with developmental stuttering, ANS is more often associated with subcortical lesions, in particular the BG, than with lesions in cortical speech and motor regions (Ludlow and Loucks, 2003; Alm, 2004).

Acquired neurogenic stuttering can occur following lesions in almost any site in the brain, either bilateral or unilateral, cortical or subcortical, left- or right-sided, focal or diffuse (Lebrun et al., 1987). Acquired stuttering can also present with concomitant aphasia. It is thus difficult to determine the localizing significance of ANS. ANS is more common in men than in women (similarly to PDS) and is also more frequently reported following left hemisphere or bilateral lesions, but ANS is a very heterogeneous disorder, and there are reports of ANS in women following right hemisphere lesion (Fleet and Heilman, 1985). ANS can occur following temporal, parietal or occipital lobe lesions (Ardila and Lopez, 1986; Grant et al., 1999; Franco et al., 2000). Alm and Risberg (2007) propose that the main mechanism causing acquired stuttering following head injury is rotational forces at the level of the midbrain and the STN causing diffuse neuronal injuries affecting several BG pathways, and that there may also be a link between ADHD and pre- or peri-natal hypoxia causing subtle biochemical changes in striatal neurones, especially intermittent hypoxia. Repeated episodes of fetal asphyxia have been shown to cause preferential damage to the striatum in sheep, with loss of medium-sized striatal GABAergic projection neurones to the GP and to the SN (Mallard et al., 1995a,b).

Ludlow et al. (1987) reported persistent ANS in 10 patients following penetrating missile wounds in the brain during wartime. The sites of lesions in this group were compared with the sites of lesions in a group of patients with missile wounds to the brain but without speech problems. The only gray matter structures that were significantly more affected in the stuttering group were the striatum and the globus pallidum. There were lesions in the caudate or lentiform nucleus in 80% of ANS subjects, suggesting a central role of the BG in ANS. There was also cerebellar damage in 50% of the ANS subjects.

Reports of acquired stuttering following lesions affecting subcortical structures including the putamen, caudate, thalamus sub-insular WM, periventricular deep WM, pons, and rostral brainstem support the hypothesis that BG are implicated in stuttering (see **Table 1**; Leenders et al., 1986; Kono et al., 1998; Shibuya et al., 1998; Carluer et al., 2000; Ciabarra et al., 2000; Fawcett, 2005).

**Table 1:** Cases of acquired neurogenic stuttering following subcortical lesions.

Reference	Pathology	Imaging	Number of cases	Side of lesion	Structures	History of stuttering	Handedness	Gender	Clinical manifestations
Fleet and Heilman (1985)	Infarct	Arteriogram	1	R	Internal carotid artery	No personal history of stuttering; but had a positive family history of developmental stuttering	RH	F	Stuttering without aphasia
Ardila and Lopez (1986)	Infarct	CT	1	R	Extensive temporal lobe lesion	Nil	RH	M	Stuttering and left hemiparesis
Leenders et al. (1986)	Calcified lesion of unknown etiology	CT and MRI F-DOPA PET	1	L	Thalamus and rostral brainstem Significantly reduced F -DOPA uptake in the left striatum (despite absence of structural lesion)	No history of stuttering. History of childhood onset progressive right hemidystonia	RH	M	Stuttering, left-sided blepharospasm, right-sided rigidity and bradykinesia (responsive to dopamine agonists)
Ludlow et al. (1987)	Missile wounds	CT	10	5 R 4 L 1 bilat	See text	No history of childhood stuttering	8 RH; 1 LH; 1 mixed	M	Chronic acquired stuttering with repetitions, prolongations, and blocks
Abe et al. (1993)	Infarct	CT and MRI	1	Bilat	Left medial midbrain and right paramedian thalami	Nil	RH	M	Stuttering (with numerous initial syllable repetitions)
Ackermann et al. (1996)	Infarct		1		Mesiofrontal cortex (SMA)				Stuttering and transcortical motor aphasia
Kono et al. (1998)	Infarct	CT and MRI	1	L	Putamen and caudate	Nil	RH	M	Stuttering and right facial palsy
Shibuya et al. (1998)	Infarct	MRI	1	Bilat	BG and periventricular deep WM	Nil	RH	M	Stuttering and L-DOPA resistant progressive pure akinesia syndrome
Van Borsel et al. (1998)	Hemorrhage	CT and MRI	1	L	SMA	Nil	RH	M	Stuttering
Grant et al. (1999)	Infarct Infarct Infarct Infarct	MRI MRI CT MRI	4	L Bilat R L	MCA infarct – left frontotemporoparietal cortex Left posterior temporal lobe and bilateral cerebellum Parietal lobe Medial occipital lobe	Nil History of childhood stuttering History of childhood stuttering Nil	RH RH LH/mixed RH	M M M M	Stuttering and right hemiparesis Non-fluent aphasia and right hemiparesis Stuttering and left facial weakness Stuttering and right hemianopia
Franco et al. (2000)	Infarct	MRI Angiogram	1	L	Precentral gyrus Stenosis left internal carotid (intracavernous portion)	Nil	RH	M	Stuttering without aphasia
Carluer et al. (2000)	Infarct	CT and MRI	1	L	Putamen, caudate, internal capsule, and adjacent WM	Nil	RH	M	Stuttering and parkinsonian symptoms
Ciabarra et al. (2000)	Infarct Infarct Infarct	MRI MRI	3	L L L	Rostromedial pons Putamen, caudate, corona radiata Corona radiata and putamen	Nil Nil Nil	RH RH LH	M F F	Stuttering and cerebellar dysfunction Stuttering and dysarthria Stuttering and mild aphasia
Hagiwara et al. (2000)	Infarct	MRI	1	R	Corpus callosum (anterior callosal artery)	Nil	RH	M	Stuttering and left hand apraxia
Mouradian et al. (2000)	CVA	MRI	1	L	Non-specific WM changes	History of resolved childhood stuttering	R	F	Stuttering and right leg weakness
Turgut et al. (2002)	Infarct	CT and MRI	1	L	Parietal cortex	Nil	RH	M	Stuttering without aphasia
Van Borsel et al. (2003b)	Infarct	CT and MRI	1	L	Ventrolateral thalamus	Nil	RH	M	Severe stuttering during propositional speech
Fawcett (2005)	Infarct	CT	1	L	BG (lacunar infarct)	Nil	LH	F	Stuttering and right-sided motor impairment

*L, left-sided lesion; R, right-sided lesion; Bilat, bilateral lesions; LH, left-handed; RH, right-handed; mixed, mixed handedness; F, female; M, male; TCMA, transcortical motor aphasia.*

Thus ANS can be heterogeneous in its speech manifestations. Van Borsel et al. (2003b) report a case of ANS following an ischaemic lesion of the left ventrolateral thalamus, with severe stuttering during propositional speech but only mild stuttering during non-confrontational speech and therefore propose that thalamic stuttering is a distinct clinical entity. Abe et al. (1993) report a case of ANS following midbrain and paramedian thalami. The patient’s ANS differed from other cases of stuttering in that it was characterized by numerous repetitions (7) of the first syllables of words at a constant rate and loudness, in a very monotonous manner. It was thus similar to palilalia, which has also been reported in a patient with infarcts in the paramedia thalami and midbrain (Yasuda et al., 1990) as well as in patients with PD. Abe et al. (1993) posited that the repetitive speech disturbance in this patient was not attributable to the extrapyramidal system but rather to projections to the SMA from the infarcted regions of the thalamus and midbrain, because the clinical features were similar to those reported for ANS patients with SMA infarcts. Ackermann et al. (1996) reported a case of ANS affecting only word-initial sounds and transcortical motor aphasia (TCMA) following an SMA infarct. They thus proposed that ANS due to SMA lesions represents a distinct clinical entity compared to ANS associated with lesions in other areas. In contrast, Van Borsel et al. (1998) reported a case of severe ANS following a left SMA hemorrhage in which there was a different clinical picture, with stuttering not limited to word-initial position and present when reading aloud and during sentence repetition. Thus lesions of the same area can give rise to different types of ANS.

### ACQUIRED STUTTERING ASSOCIATED WITH THE THALAMUS

Among the most articulate proponents of a possible thalamic contribution to language and speech are Penfield and Roberts (1959) who assessed such functions in patients who suffered from focal cerebral seizures, and who underwent temporal lobe excisions involving various amounts of neural tissue. They consequently proposed, as a speech hypothesis, “that the functions of all three cortical speech areas in man are coordinated by projections of each, to parts of the thalamus, and by means of these circuits the elaboration of speech is carried out.” Our knowledge about the role of thalamus in ANS has increased with the advent of stereotactic neurosurgery. Hassler (1966) noted that stimulation of the ventrolateral thalamus produces acceleration or blocking of vocalization. Samra et al. (1969) in an extensive study of the anatomical location of the lesions in the brains of 27 patients with PD who had undergone thalamic surgery noted that “the presence of dysfluencies may depend more on the motor cortex-ventrolateral thalamus modulation than to thalamic influences in general (…). Consequently bilateral destruction of this thalamic zone may account for the more obvious and long-standing speech phenomena of hesitations, blocking, or increase of rate of speaking (i.e., palilalia).” Andy and Bhatnagar (1992) report four patients with mesothalamus dysfunction and a history of chronic pain, absence seizures, and dyskinesias who went on to develop acquired stuttering as part of a larger syndrome complex. Chronic implantation of stimulating electrodes in the left centromedian nucleus of the thalamus was performed as a last resort treatment for the patients’ chronic pain and other symptoms. All four patients had spontaneously occurring abnormal EEG discharges in the mesothalamus. Their stuttering lacked secondary behaviors and failed to show adaptation, but featured numerous blocks. Unipolar self-stimulation of the CM nucleus attenuated the abnormal EEG discharges and improved the stuttering, in addition to the chronic pain and other symptoms. All four patients remained stutter-free post-operatively (and had ∼90% improvement in other symptoms).

Schaltenbrand (1975) also noted that stimulation of the thalamus and of the corpus callosum during stereotactic surgeries to treat epilepsy, chronic pain and dyskinesias had effects on speech. Stimulation of the anterior corpus callosum with the stereotactic needle silenced speech, stimulation of the posterior corpus callosum caused confused thinking and interrupted speech. Stimulation of the posterior and ventro-oral thalamus resulted in alterations in articulation and in interruption of speech. Stimulation of the deep thalamus gave rise to various kinds of shouts and utterances. The effects reported were predominantly associated with stimulation of the dominant hemisphere. The pattern of this evoked compulsory speech resembled that of stuttering and palilalia.

Mechanical perturbation of the thalamus (advancing a 1 mm diameter electrode 2 mm in the post-eroventromedial thalamus) intraoperatively in a patient having lesion surgery for chronic pain was found to cause repetitive speech dysfluencies similar to stuttering (Andy and Bhatnagar, 1991). Electrophysiological recording showed concurrent abnormal discharge from the part of the thalamus being perturbed. There are also reports of alleviation of stuttering upon electrical stimulation of the same site in the thalamus (Bhatnagar and Andy, 1989). These observations suggest that the mesothalamus is part of a speech-regulating corticomesothalamic feedback pathway.

Anomia and preservation can be evoked by electrical stimulation of the left ventrolateral thalamus (specifically the medial central portion; Ojemann and Ward, 1971), and stimulation of an adjacent area of the (pulvinar and inferior) ventrolateral thalamus in right handed patients results in anomic responses (Ojemann et al., 1968), suggesting a speech integrating center in the lateral thalamus.

### THE BASAL GANGLIA AND SPEECH PATHWAYS IN STUTTERING

The neural pathways of the BG remain incompletely understood, but they are known to be involved in the selection of competing voluntary motor programs (generated by the cortex and cerebellum), disinhibiting one selected motor program and simultaneously inhibiting all other competing motor programs in order to allow the execution of voluntary movements (Mink, 1996). Thus the BG do not themselves generate movements, but rather play a central role in the selection of competing voluntary movement patterns, inhibiting competing motor programs that would otherwise prevent execution of the desired movement. Degenerative disease of the BG is known to cause a number of movement disorders characterized by slow movements, involuntary muscle activity, or abnormal postures, including PD, dystonia, and tremor of various etiologies. There is evidence that stuttering may be a movement disorder of speech involving BG dysfunction (Alm, 2004; Max et al., 2004b).

Jürgens (2002) posited that there is a cerebello-thalamo-cortical pathway implicated in normal speech production in humans, based on lesion studies in humans and functional and structural studies in the macaque monkey and other primates. Speech is severely affected following lesions to the cerebellum (Ackermann and Ziegler, 1991) and to the ventrolateral thalamus and, as mentioned above, electrical stimulation of areas of the thalamus can produce vocalizations in humans (Schaltenbrand, 1975; Lechtenberg and Gilman, 1978). PET and fMRI studies have shown bilateral activation in the ventrolateral thalamus and the cerebellum during speech and singing tasks (Petersen et al., 1988; Herholz et al., 1994; Hirano et al., 1996; Price et al., 1996; Perry et al., 1999; Bookheimer et al., 2000). Medial parts of the ventrolateral thalamus contain facial muscle representations and show increased activity during vocalization (Vitek et al., 1994; Farley, 1997). The ventrolateral thalamus has projections to M1, to Broca’s area and to the SMA (Nakano et al., 1992; Rouiller et al., 1994).

Alm (2004) proposes that there is a medial BG-SMA route and a lateral cerebellar-lateral pre-motor cortex (including Broca’s area) route, and that in PDS there is dysfunction in the BG-cortical route and compensatory overactivation of the cerebellar-cortical route. This could be consistent with the evidence of cerebellar overactivity in PWS reported by Brown et al. (2005). Structural equation modeling (SEM) also provides evidence of altered connectivity in the basal ganglia-thalamo-cortical circuit in PWS (Lu et al., 2009; Civier et al., 2013).

The role of BG during dysfluent speech has been extensively described (Alm, 2005). The increased neural activation on event-related fMRI in the putamen bilaterally in stutterers following fluency-inducing therapy suggests that the putamen is implicated in speech motor control in PDS (Neumann et al., 2003, 2005). However, this increased activation in the putamen did not persist at 2 year follow-up, unlike the therapy-associated increased activation found in other regions, including limbic areas, bilateral temporal cortex, and right parietal and frontal cortex.

An fMRI study of reading tasks in PWS before and after fluency-inducing therapy showed a statistically significant correlation between stuttering severity and BG activity, lending further support to the BG hypothesis of stuttering (Giraud et al., 2008). Pre-treatment (*n* = 16), there was a significant (*p* < 0.001) positive correlation between stuttering severity and bilateral caudate nucleus activity and activity in left medial superior posterior parietal/post-central regions (BA 4/5/7). There was also a negative correlation of stuttering severity with activity in the left SN. Following therapy (*n* = 9), this pattern of activation was lost and there was a correlation between pre-treatment stuttering severity and a small area of left caudate activation, but this failed to reach significance. There was no significant correlation between increase in caudate activity and improvement in fluency with therapy, as would be expected if the caudate were implicated in compensation. Giraud et al. (2008) proposed a functional model of stuttering in which structural abnormalities affecting flow of information from Broca’s area to the motor cortex engender BG dysfunction. Their model is based on cortico-striato-cortico loops and models of dysfunction in these loops in BG disorders such as PD and dystonia (see figure, p. 197, Giraud et al., 2008).

### DYSFLUENCY IN PARKINSON’S DISEASE

The term “palilalie” (from the greek “palin” again and “lalia” speech) in the context of acquired neurological disease was first used by Souques (1908) one of the talented House Officers of Charcot Walusinski (2011). Souques reported on a particular disturbance of language in a patient with stroke leading to left-sided hemiplegia, which presented as compulsive repetition of semantically adequate answers to the examiners’ questions. This symptom termed palilalia by Souques was also observed in post-encephalic Parkinsonism. Post-mortem examinations have suggested lesions of the striatum as the anatomical substratum of the disease (Critchley, 1927). In the cases reported so far, palilalia was either constantly present or varied in degree; it occurred both in spontaneous speech and in replying to questions, but not often when reading or reciting a well known text; the number of repetitions usually range between four and eight (Ackermann et al., 1989); reiterations comprised syllables, words or sentences. Often the repetitions tend to be uttered with increasing rapidity and decreased loudness (Critchley, 1927; Ackermann et al., 1989).

This complex speech disturbance in PD can resemble stuttering, lending further support to a pathophysiological role of the BG in ANS. Benke et al. (2000) proposed that repetitive speech phenomena in PD patients can be divided into two types, the first resembling palilalia (with hyperfluent repetitions, fast utterances, increased speech rate and often blurred or murmured due to articulation that is poor or decreasing in loudness) and the second more similar to PDS, with dysfluent, prolonged, relatively well articulated speech, in a constant rate and loudness. In their study of 53 patients with idiopathic PD, 15 had repetitive speech phenomena. In these 15 patients, both types of dysfluency were present, with constant distribution across speech tasks. They noted that the repetitive speech phenomena were more noticeable in patients with longer disease duration and fluctuating motor response to levodopa (54.3% of the advanced patients). There was no significant difference in repetitive speech phenomena in the on- and off- medication states. They conclude that the palilalia was therefore unlikely to be a type of levodopa-induced hyperkinesia of speech. Conversely Ackermann et al. (1989), described a patient with marked palilalia when on-medication only on repetition-type tasks and not on spontaneous speech and they conclude that it was a sign of medication-induced hyperkinesia.

## STUTTERING IMPROVING OR WORSENING AFTER DEEP BRAIN STIMULATION (DBS) SURGERY

Deep brain stimulation (DBS) is an established surgical therapy for the management of BG motor disorders such as PD, dystonia and tremor, and the safety and efficacy of DBS in motor disorders has led to its use in an expanding range of other motor and psychological disorders such as Gilles de la Tourette syndrome, obsessive- compulsive disorder and severe depression. DBS affords a unique opportunity to study the pathophysiology of BG disorders and has advanced understanding of BG pathways. Cases of stuttering worsening or improving following implantation of stimulating electrodes into BG nuclei for other indications, and more general speech changes following DBS, shed further light on the role of the BG in stuttering (**Table 2**).

**Table 2:** Cases of improvement or worsening in stuttering following DBS.

Reference	Number of cases	DBS target lesions	Indication for DBS	History of stuttering	Handedness	Gender	Clinical manifestations
Moretti et al. (2003)	1	Bilateral STN	PD	No prior history of stuttering	RH	M	Motor scores greatly improved in the stimulation ON condition, but difficulty initiating speech, repetitions of initial syllables or phonemes and difficulty proceeding. Circumlocutions and preservations and a decreased speech rate
Burghaus et al. (2006)	1	bilateral STN	PD	Childhood stuttering, with onset at the age of 9, improvement in adolescence but then marked worsening after onset of PD. Also PD-related hypophonia and hypokinetic dysarthria	RH	M	Improvement in Parkinson-related hypophonia but worsening of stuttering following surgery
Walker et al. (2009)	1	Unilateral (left) STN	PD	Acquired stuttering associated with PD (no personal or family history of developmental stuttering)	RH	M	Statistically significant improvement in stuttering in the neurostimulation on state compared to the off state, irrespective of whether on or off dopaminergic medications
Nebel et al. (2009)	2	Bilateral GPi Bilateral GPi	DYT1 mutation positive generalized dystonia DYT1 negative segmental dystonia of the neck, trunk and upper limbs	No history of childhood stuttering. Speech was relatively unaffected by dystonia No history of childhood stuttering	RH	M M	Significant improvement in motor function following implantation. Stuttering that appeared gradually and was unrelated to changes in stimulation parameters In post-operative programming, a change in a stimulation contact improved his motor symptoms but also provoked dysarthria and dysfluency, characterized by blocks, iterations, and intermittent rushes of unintelligible utterances (abnormal speech timing or hastening)
Toft and Dietrichs (2011)	2	Bilateral STN Bilateral STN	PD PD	Acquired stuttering associated with PD Childhood stuttering that had re-emerged during course of PD progression	RH	M	Worsening of stuttering following surgery

*PD, Parksinson’s disease; STN, subthalamic nucleus; GPi, globus pallidus internus; M, male; RH, right-handed*.

Walker et al. (2009) report an unusual case of PD-associated acquired stuttering, which improved following unilateral STN DBS. This is in contrast to other cases, where stuttering worsens or reappears subsequent to STN DBS.

Toft and Dietrichs (2011) reported two cases of PD patients who underwent bilateral STN DBS. One patient had acquired PD-associated stuttering with that worsening followed surgery, and the other had childhood stuttering that had re-emerged during course of PD progression and which worsened following surgery.

Moretti et al. (2003) report a case of stuttering appearing following bilateral DBS STN for PD. Following implantation, the stimulation ON condition was associated with greatly improved motor scores but also with newly acquired stuttering, which persisted at follow-up.

Burghaus et al. (2006) reported a case of stuttering worsening subsequent to bilateral STN DBS for PD. The patient had childhood stuttering, which improved in adolescence but then markedly worsened after onset of PD, and also Parkinson-related speech changes (hypophonia and hypokinetic dysarthria). Following surgery, there was an improvement in his Parkinson-related hypophonia but a worsening of stuttering, with increased frequency of blocks, prolongations and syllable repetitions and also of facial grimaces. This worsening in stuttering was marked with bilateral stimulation, but there was no significant effect of unilateral stimulation on stuttering severity. Stimulation-induced motor improvement was associated with worsening of stuttering. There were no tetanic muscle contractions or other side effects to suggest that the speech disturbances were the result of current spread to the internal capsule. PET was performed in the off-drug state comparing on- and off-DBS states during resting conditions and during a speech task. In the DBS-off condition during the speech task, there was increased rCBF in the right posterior STG (Wernicke/BA29), in the left lower frontal gyrus (Broca’s area) and the adjacent anterior insula (BA44) and in the left anterior cingulum (BA24). Further increases in rCBF occurred in caudal M1 (BA4), SMA (BA6), and the dorsolateral prefrontal cortex (BA6/9) bilaterally and in the right cerebellar hemisphere. In the DBS on condition, there was increased rCBF in the left rostral SMA and in M1 on the right, in addition to in the anterior cingulate and the cerebellar hemispheres bilaterally. rCBF in the anterior insula and Broca’s area was increased compared to during the DBS off condition. There were no significant changes in rCBF in Wernicke’s area (left posterior STG) in the DBS on state.

Stuttering can also occur following GPi DBS for dystonia, although the most commonly reported adverse effect of GPi DBS is dysarthria, with a prevalence of up to 12% (Kupsch et al., 2006).

Nebel et al. (2009) reported two cases of stuttering following GPi DBS (of their series of 67 patients), which was distinct from dysarthria. The first patient had DYT1 mutation positive severe generalized dystonia but his speech was relatively unaffected. He had significant improvement in motor function following bilateral GPi-DBS implantation but new onset stuttering appeared gradually. The stuttering was apparently unrelated to changes in stimulation parameters and progressively worsened (8 months post-operatively his speech was unintelligible to a speech therapist). He did not have any dysarthria, palilalia, accessory motor symptoms, or anxiety. The second patient underwent bilateral GPI DBS for DYT1 negative segmental dystonia of the neck, trunk and upper limbs. In post-operative programming, a change in a stimulation contact improved his motor symptoms but also provoked dysarthria and stuttering.

## DOPAMINE AND STUTTERING

According to the dopamine excess hypothesis of stuttering, there is a hyperdopaminergic state in PDS (Wu et al., 1997; Anderson et al., 1999). Neuropharmacological studies have failed to provide unequivocal evidence of this, because although L-dopa can increase dysfluency in PD (Anderson et al., 1999; Louis et al., 2001) and despite reports of stuttering improving with dopamine antagonists such as haloperidol, risperidone, and olanzapine (Healy, 1974; Murray et al., 1977; Burns et al., 1978; Maguire et al., 2000, 2004), Goberman and Blomgren (2003) reported no significant difference in dysfluency in PD patients in low and high dopamine states.

However, a case control study of the allelic frequencies of five single nucleotide polymorphisms (SNPs) in two dopaminergic genes lends support to the dopamine excess hypothesis (Lan et al., 2009). They report a significantly higher frequency of C alleles than T alleles in stutterers compared to controls at the rs6277 site of the DRD2 gene. Hirvonen et al. (2004) found evidence of an association between the CC phenotype of the rs6277 SNP of the DRD2 gene and decreased D2 receptor binding and increased synaptic cleft dopamine density. This would be consistent with a hyperdopaminergic state in stuttering, and with the hypothesis of increased D2 to D1 receptor ratio in the striatum in developmental stuttering proposed by Alm (2004).

### NEUROIMAGING OF GLUCOSE AND DOPAMINE NEURAL METABOLISM IN STUTTERING

Stuttering can be decreased using dopamine antagonists (see below). Wu et al. (1995) reported decreased cortical and subcortical glucose metabolic rates in stutterers compared to controls, which could be due to excess dopamine activity as amphetamine (a dopamine agonist) and cocaine (a dopamine reuptake inhibitor) inhibit regional cerebral glucose metabolic activity. In an fluorodeoxyglucose (FDG) PET study of solo and choral reading in four PWS and four controls, all right-handed, Wu et al. (1995) found both a state- (stuttering versus fluent reading, i.e., solo versus chorus reading tasks) and trait- (stutterers versus controls) dependent decrease in glucose uptake in cortical and subcortical areas in the stutterers (*p*< 0.05).

There was a trait-related state-dependent decrease in glucose uptake in the superior frontal cortex (BA9), in Wernicke’s areas (BA39, BA40), Broca’s area (BA45), in the posterior cingulate (BA23), in the prefrontal cortex (BA10), in the deep frontal orbital cortex (BA11) and in the medial cerebellum. These areas of hypometabolism can be broadly divided into four categories: left language areas (Wernicke’s and Broca’s), higher order association areas (superior frontal cortex and prefrontal cortex), the left cerebellum, and limbic areas (deep frontal orbital cortex and the posterior cingulate).

Overall, no region had greater glucose uptake during stuttering compared to choral reading or during stuttering compared to reading in normal controls. Comparison of choral reading in stutterers (which induced fluency) with reading in controls revealed two key differences, both in the BG. The largest difference between stutterers and controls was in the left caudate, which showed two areas of reduced glucose uptake during solo reading in stutterers compared to solo reading in controls. The left caudate was ∼50% less active in stutters versus controls for the stuttering state, and the caudate did not show any normalizing increase in glucose uptake during choral reading in stutterers. In the SN/ventral tegmental area, there was markedly increased glucose uptake in the stutterers during the choral reading task. This non trait-related state-dependent change in metabolism suggests an increased rate of neuronal firing in the SN in the induced fluency state.

Thus there was a permanent hypometabolism of the left caudate that may be a trait-related marker in PWS, and reversible hypometabolism in left language areas and higher association areas. There was decreased cerebellar glucose uptake in the stutterers during the solo reading task, but the metabolism of the right cerebellum increased to be comparable to that of normal controls during choral reading in stutterers. Lastly, increased limbic metabolism in stutterers during fluent choral speech may correlate with reduced speech-associated anxiety. It should be noted that this study did not include the SMA because image acquisitions did not extend high enough.

In a fluorodopa PET study of three subjects with PDS and six controls, all right-handed, Wu et al. (1997) reported results consistent with the dopamine excess theory of stuttering. FDOPA is a dopamine precursor used as a means of measuring the rate of dopamine synthesis in the brain (Barrio et al., 1997). Wu et al. (1997) found that compared to controls, stutterers had nearly three times increased FDOPA uptake activity in the right ventral medical prefrontal cortex (BA32, *p* < 0.01) and left caudate tail (*p*< 0.05). Stutterers also had a greater than 100% increase in FDOPA uptake in limbic structures including the left extended amygdala, the left insular cortex and the right deep orbital cortex (*p* < 0.05), and also in auditory cortex (BA22, *p*< 0.05). Overall, the greatest increased in FDOPA uptake activity in stutterers was in ventral limbic areas, which Wu et al. (1995) found to have decreased metabolic activity and which they proposed are involved in neural circuits of stuttering. The medial prefrontal cortex receives extensive dopaminergic innervations and has functional connections to the SMA (Bunney and Aghajanian, 1977; Tassin et al., 1977; Chiodo et al., 1984; Thierry et al., 1988; Weinberger et al., 1988; Bertolucci-D’Angio et al., 1990; Cenci et al., 1992). Furthermore, the medial prefrontal cortex has been identified as a vocalization center in primates (Jürgens and Müller-Preuss, 1977; Jürgens and Pratt, 1979; Jürgens, 1986). Wu et al. (1997) proposed that their findings may indicate abnormal overactivity in mesocortical dopamine tracts. Dopaminergic tracts also project to temporal cortical regions (De Keyser et al., 1989). The results of Wu et al. ’s (1997) study are of limited power due to the small sample size, but they nonetheless suggest an association between increased dopamine activity in brain regions implicated in speech production and stuttering and thus lend credence to the dopamine excess theory of stuttering.

### D2 RECEPTOR ANTAGONISTS AND STUTTERING

There are reports of haloperidol-associated improvement in stuttering (Healy, 1974; Burns et al., 1978), and of improvement or complete resolution of ANS when treated with paroxetine, a potent and selective serotonin reuptake inhibitor (SSRI; Schreiber and Pick, 1997; Costa and Kroll, 2000; Boldrini et al., 2003). It has been proposed that there are interactions between serotonergic and dopaminergic systems in the forebrain (specifically in the SMA, which has connections to the BG), and that paroxetine may improve stuttering via a serotonin-mediated indirect antidopaminergic effect in such patients. Turgut et al. (2002) report a case of ANS subsequent to focal left parietal infarct, which resolved completely with paroxetine therapy. By contrast, there are reports of SSRIs including sertraline and fluoxetine causing stuttering (Guthrie and Grunhaus, 1990; Christensen et al., 1996). SSRIs are not a homogenous class of drugs (Sokolowski and Seiden, 1999), so it possible that effects on serotonergic and dopaminergic systems differ between agents.

Ecstasy (MDMA) has antiparkinsonian effects in primates, possibly via a serotonergic mechanism by agonist effect on 5HT1a or 5HT1b receptors (Iravani et al., 2003). The anti-parkinsonian effect of ecstasy in primates is completely blocked by fluroxamine, a SSRI. A case of Parkinsonism associated with MDMA use in humans has also been reported (Kuniyoshi and Jankovic, 2003).

There are reports of stuttering improving with amphetamine treatment (Fish and Bowling, 1962, 1965). There is evidence that amphetamine results in a long lasting decrease in the number of D1 and D2 receptors available in the striatum (due to cytoplasmic internalization of receptors; Dumartin et al., 1998; Ginovart et al., 1999; Sun et al., 2003). The down-regulatory effect of amphetamine on striatal dopamine receptors may be relatively greater for D2 receptors versus D1 receptors (Gifford et al., 2000), which would be consistent with the hypothesis of a relationship between high D2 receptor density in the putamen and stuttering proposed by Alm (2004).

Cases of stimulant-induced stuttering have also been reported, and may be attributable to increased dopaminergic neurotransmission (Burd and Kerbeshian, 1991). Stimulants are thought to affect dopaminergic and noradrenergic systems in children with ADHD, and can worsen tic symptoms and even trigger tic disorders (Lowe et al., 1982). There are reports of theophylline-induced stuttering in children and adults and it has been proposed that theophylline engenders stuttering by disturbing dopaminergic neurotransmission (indirectly via inhibition of GABA and adenosine receptors), with a hyperdopaminergic effect that is greatest in the BG (McCarthy, 1981; Rosenfield et al., 1994; Gérard et al., 1998; Movsessian, 2005).

## STUTTERING AS A FORM OF DYSTONIA

It is possible that stuttering represents a form of focal segmental dystonia of the orofacial muscles (Alm, 2005). The involuntary movements seen in stuttering are similar to those in dystonia, and sensitivity to emotional stress is common to stuttering and to focal dystonias (Kiziltan and Akalin, 1996). A family history of stuttering is more common in patients with idiopathic torsion dystonia than in the general population (Fletcher et al., 1991). There is also evidence of a hyperdopaminergic state in both disorders. Kiziltan and Akalin (1996) propose that stuttering is a focal/segmental action dystonia. Dystonia can frequently result from focal lesions of the BG (Bhatia and Marsden, 1994; Naumann et al., 1996). Others argue that the presence of involuntary movements similar to those seen in BG movement disorders and complex motor tics in patients with PDS suggests a common pathophysiology for tics and stuttering (Mulligan et al., 2003).

## CONCLUSION AND FUTURE PERSPECTIVES

The etiology and pathophysiology of stuttering remain poorly. Stuttering is a disorder associated with significant psychological burden and social stigma, and work toward achieving successful therapies has been focusing on its psychological or psychodynamic causes. The increased recognition of a structural or functional neurological cause can render stuttering potentially amenable to surgical or medical intervention. Further research on the cortical and subcortical anatomical and functional changes in stuttering is needed. In this study, we have reviewed evidence demonstrating that dysfunction of the BG and of their cortical targets are a likely pathomechanism underlying stuttering.

## Conflict of Interest Statement

The authors declare that the research was conducted in the absence of any commercial or financial relationships that could be construed as a potential conflict of interest.
